# Response Properties of Interneurons and Pyramidal Neurons in Macaque MSTd and VPS Areas During Self-Motion

**DOI:** 10.3389/fncir.2018.00105

**Published:** 2018-11-23

**Authors:** Yingying Zhang, Shasha Li, Danqing Jiang, Aihua Chen

**Affiliations:** Key Laboratory of Brain Functional Genomics (Ministry of Education), East China Normal University, Shanghai, China

**Keywords:** interneuron, pyramidal neuron, self-motion, visual, vestibular, MSTd, VPS

## Abstract

To perceive self-motion perception, the brain needs to integrate multi-modal sensory signals such as visual, vestibular and proprioceptive cues. Self-motion perception is very complex and involves multi candidate areas. Previous studies related to self-motion perception during passive motion have revealed that some of the areas show selective response to different directions for both visual (optic flow) and vestibular stimuli, such as the dorsal subdivision of the medial superior temporal area (MSTd) and the visual posterior sylvian fissure (VPS), although MSTd is dominated by visual signals and VPS is dominated by vestibular signals. However, none of studies related to self-motion perception have distinguished the different neuron types with distinct neuronal properties in cortical microcircuitry, which limited our understanding of the local circuits for self-motion perception. In the current study, we classified the recorded MSTd and VPS neurons into putative pyramidal neurons and putative interneurons based on the extracellular action potential waveforms and spontaneous firing rates. We found that: (1) the putative interneurons exhibited obviously broader direction tuning than putative pyramidal neurons in response to their dominant (visual for MSTd; vestibular for VPS) stimulation type; (2) either in visual or vestibular condition, the putative interneurons were more responsive but with larger variability than the putative pyramidal neurons for both MSTd and VPS areas; and (3) the timing of vestibular and visual peak directional tuning was earlier in the putative interneurons than that of the putative pyramidal neurons for both MSTd and VPS areas. Based on these findings we speculated that, within the microcircuitry, several adjacent putative interneurons with broad direction tuning receive earlier strong but variable signals, which might act feedforward input to shape the direction tuning of the target putative pyramidal neuron, but each interneuron may participate in several microcircuitries, targeting different output neurons.

## Introduction

In everyday life, perception of self-motion is essential for navigation, spatial orientation and motor control. It is vital for our living and survival. To perceive self-motion, the brain needs to integrate information from visual, vestibular, auditory, kinesthetic and somatosensory sensors (Warren, 1990; Gottfried and Dolan, 2003; Pettorossi and Schieppati, 2014). Previous researches in passive motion conditions have revealed that both visual (optic flow across the retina) and vestibular (sensed by otolith organs when the head is translating in space) provide powerful cues for the heading (here, referred as the direction of the self-motion) perception in human self-motion (Telford et al., 1995; Ohmi, 1996; Harris et al., 2000; Bertin and Berthoz, 2004; Butler et al., 2010). Meanwhile physiological studies have proved that many neurons in several cortical areas, including dorsal subdivision of the medial superior temporal (MSTd) area (Britten and van Wezel, 1998, 2002; Duffy, 1998; Bremmer et al., 1999; Gu et al., 2006; Britten, 2008; Fetsch et al., 2010; Angelaki et al., 2011) and visual posterior sylvian fissure (VPS) area (Jones and Burton, 1976; Guldin et al., 1992; Guldin and Grüsser, 1998; Dicke et al., 2008; Chen et al., 2011a), show selectivity for heading in response to both visual and vestibular self-motion stimuli, although the responses to visual and vestibular stimuli varies across different cortical areas. MSTd area shows visual-dominant heading tuning while VPS area shows vestibular-dominant heading tuning (Chen et al., 2011b). These studies have enriched our knowledge about the self-motion perception in passive motion conditions and advanced our understanding of the underlying neural mechanism.

However, none of these self-motion related studies considered the diversity of cell types within local microcircuits, which are known to exist in cortex (Ramon and Cajal, 1899) and have been studied widely *in vitro* (Peters and Jones, 1984; Toledo-Rodriguez et al., 2003; Markram et al., 2004). Intracellular recordings in cortical slices have shown that action potentials produced by GABAergic inhibitory interneurons were shorter in duration than that produced by glutamatergic excitatory pyramidal cells (McCormick et al., 1985; Connors and Gutnick, 1990; Nowak et al., 2003). Since intracellular characteristics determine the extracellular waveform (Henze et al., 2000; Gold et al., 2006), duration of extracellular spike is usually used as a basic parameter in distinguishing inhibitory interneurons (narrow-spiking, NS) from pyramidal neurons (broad-spiking, BS). Based on the waveform, a number of recent studies have begun to explore the role of these two classes of neurons in cortical microcircuits in visual cortex (Gur et al., 1999; Mitchell et al., 2007; Chen et al., 2008; Woloszyn and Sheinberg, 2012), somatosensory cortex (Mountcastle et al., 1969), auditory cortex (Tsunada et al., 2012), motor cortex (Kaufman et al., 2010, 2013), parietal cortex (Yokoi and Komatsu, 2010) prefrontal cortex (Wilson et al., 1994; Rao et al., 1999; Constantinidis and Goldman-Rakic, 2002; Diester and Nieder, 2008; Hussar and Pasternak, 2009, 2012; Johnston et al., 2009), and the hippocampus (Csicsvari et al., 1999; Kuang et al., 2010), and have demonstrated a differential functional role of interneurons and pyramidal neurons. For instance, in macaque area V4, putative interneurons are modulated stronger than pyramidal neurons by attention during multiple-object tracking task (Mitchell et al., 2007). Thus, it is imperative to comprehend how inhibitory interneurons and pyramidal neurons play different roles during self-motion perception? Is the difference of the roles reflected by the spatio-temporal direction tuning properties between interneurons and pyramidal neurons? To test these hypotheses, we classified the recorded neurons into BS neurons (putative pyramidal neurons) and NS neurons (putative interneurons) based on their known differences in extracellular action potential waveform, and compared their heading response properties based on visual and vestibular stimuli.

## Materials and Methods

### Subjects

Physiological data were obtained from five adult male monkeys (Macaca mulatta), whose weights ranged from 6 kg to 10 kg. A circular plastic ring was chronically implanted on the head of each monkey for head restraint; a magnetic field coil was also chronically implanted on the sclera of each monkey for monitoring eye-movement; and a plastic grid was fixed in the circular ring for positioning the electrode (for details, see Gu et al., 2006). After sufficient recovery, animals were trained using standard operant conditioning to receive liquid reward for fixation to visual targets. This study was carried out in accordance with the recommendations of the Institutional Animal Care and Use Committee at East China Normal University. The protocol was approved by the Institutional Animal Care and Use Committee at East China Normal University.

### Apparatus and Motion Stimuli

**Figure 1:**
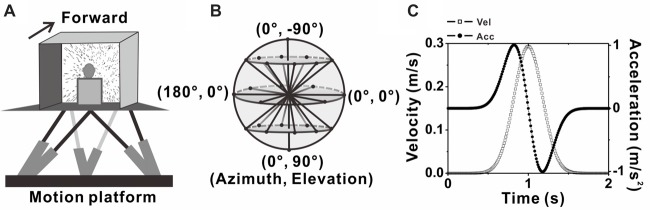
Experimental setup and transient translation stimuli. **(A)** Schematic illustration of the virtual reality apparatus. Monkeys were seated on a motion platform, with six degrees of freedom, which provided the vestibular stimulus. Visual stimulus (optic flow) was displayed on the screen located in front of the monkey. **(B)** Illustration of the 26 movement trajectories used to measure 3D heading tuning curve, corresponding to all combinations of azimuth and elevation, in 45° increments from a sphere. **(C)** Motion Profile. All movements had a 2 s duration, originated from the center position, and had a Gaussian velocity profile (peak of 0.3m/s, dashed-square line) with a corresponding biphasic linear acceleration profile (peak of ±1 m/s^2^, dashed-dot line).

A virtual reality system including a motion platform (6DOF2000E; Moog, East Aurora, NY, USA) and a monitor mounted on that was used to simulate the vestibular and visual stimulation during self-motion. During experiments, the monkey was sitting in a chair which is mounted on the platform. The monkey’s head was fixed and was kept facing the center of the screen (Figure 1A). Vestibular stimuli were delivered by translation of the platform, and visual stimuli, simulated the identical translational self-motion through movements of random dots (optical flow) in a virtual 3D space of 100 cm wide, 100 cm tall, and 40 cm deep, were programmed in OpenGL and presented on the monitor (PHILIPS BDL4225, Royal Philips, Amsterdam, Netherlands). A magnetic field coil frame moved together with the animal as it was mounted on the platform. For more details, refer to previous studies (Gu et al., 2006; Chen et al., 2011a,b).

### Experimental Protocol

Two stimulus conditions, the vestibular condition and visual condition were used in the present study. In each condition, there are 26 evenly sampled translation directions originated from the center of the sphere to the surface of the sphere, including all combinations of eight different azimuth angles from 0° to 315° in increments of 45° and three different elevation angles: 0° and ± 45° (elevation 0 indicates the horizontal plane), along with two another elevation angles: −90° and 90° corresponding to the upward and downward movement directions respectively (Figure 1B). Movement along one of the 26 directions lasted 2 s and its velocity varied with a Gaussian profile. The peak velocity was 30 cm/s and the peak acceleration was about 0.1 g (~0.98 m/s^2^; Figure 1C), thus the displace amplitude is 13 cm in total. In vestibular condition, the monkey was moved by translation of the platform in the absence of optical flow. In visual condition, only optic flow simulating self-motion through the cloud of random dots was presented on the monitor while the platform was kept stationary. All directions under both conditions were interleaved and delivered randomly in one block of trials. In each trial, the animal has to fix its eyes on a fixation point of 0.2° in diameter in the center of the monitor for 200 ms before stimulus onset (fixation windows were restricted to 1.5° ×1.5° of visual angle) and was rewarded with a drop of juice at the end of the trial for keeping fixation during the presentation of the stimulus (only vestibular or visual stimulus was given in each trail). Trials were aborted and data discarded when the animal broke fixation at any time during the stimulation.

### Electrophysiological Recordings

Extracellular recordings from single neuron were performed using tungsten microelectrodes. The tip diameter of the electrode is 3 μm and the impedance is 1–2 MΩ at 1 kHz (Frederick Haer Company, Bowdoin, ME, USA). Through a transdural guide tube, the microelectrode was moving downward into the cortex driven by a hydraulic microdrive (Frederick Haer Company, Bowdoin, ME, USA). Electrical voltage signals from neurons were collected, amplified, filtered (400–5,000 Hz), and isolated with a metal microelectrode preamplifier (Bak Electronics, Mount Airy, MD, USA). The TEMPO system (Reflective Computing, Olympia, WA, USA) was used to control the experiment protocol and data acquisition including sending synchronous event signals to the recoding system (CED Power 1401; Cambridge Electronic Design, Cambridge, UK). The spike times and all behavioral events were sampled at 1 kHz in TEMPO system, while the raw neural signals were recorded at a rate of 25 kHz by CED. All these data were saved for off-line spike sorting. Area MSTd and VPS were identified by a combination of magnetic resonance imaging scans, stereotaxic coordinates, white/gray matter transitions, and physiological response properties, as described in detail previously (Gu et al., 2006; Chen et al., 2011a,b). Area MSTd is centered about 15 mm lateral to the midline and ~3–6 mm posterior to the interaural plane, and area VPS is located posterior to PIVC and extends approximately 4–5 mm anterior to posterior, which were consistent with previous studies (Gu et al., 2006; Chen et al., 2011a).

### Data Analysis

All the data analyses in the present study were performed using custom designed software running in Matlab (Mathworks, Natick, MA, USA). Isolated spike waveforms of every single neuron were obtained by offline spike sorting using Spike2 software (Cambridge Electronic Design, Cambridge, UK), from the raw data recorded by CED.

#### Neuron Classification

**Figure 2:**
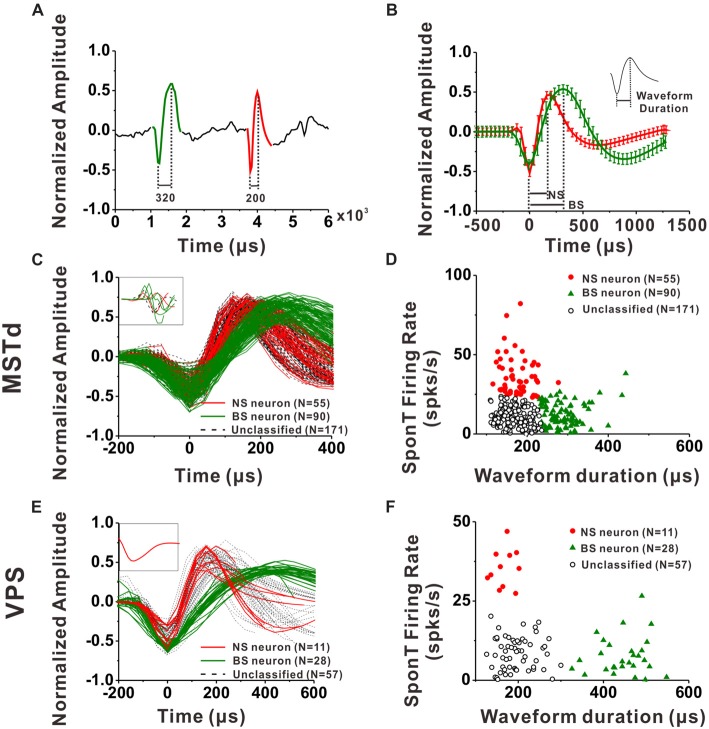
Classification of narrow-spiking (NS) and broad-spiking (BS) neurons. **(A)** Original spike waveforms of two distinct neurons isolated from simultaneous recordings of one electrode. The trough to peak durations of these two neurons are 200 μs (red, NS) and 320 μs (green, BS), respectively. **(B)** Average spike waveforms (mean ± SD, normalized by dividing the difference between the peak value and the trough value) of two distinct neurons isolated from simultaneous recordings of one electrode. The two waveforms were aligned by their troughs. The trough to peak durations of these two neurons are 188 μs (red, NS) and 313 μs (green, BS) respectively. Inserted is the illustration of trough to peak duration of a standard waveform. **(C)** The average waveforms of every neuron in the group of dorsal subdivision of the medial superior temporal (MSTd) neurons. The waveforms of eight neurons without general biphasic shape are inserted in the top left corner. **(D)** Classification results of all the isolated MSTd neurons using K-means clustering analysis running on the distribution of spike durations and SponTs. **(E)** The average waveform of every neuron in the group of visual posterior sylvian fissure (VPS) neurons. The waveforms of the discarded neuron are inserted in the top left corner. **(F)** Classification results of all the isolated VPS neurons using K-means clustering analysis running on the distribution of spike durations and SponTs.

For each neuron, all spike waveforms were spline interpolated to give a resolution of 0.2 μs and then averaged. The spike duration was defined as the time from the negative trough to the succeeding positive peak of this average waveform. It has been demonstrated by intracellular studies that pyramidal neurons are regular-spiking (RS) neurons while inhibitory interneurons are fast spiking (FS) neurons, and one striking difference between these two kinds of neurons is that the extracellular waveform of RS neurons have longer and shallower peak following the initial trough (McCormick et al., 1985; Henze et al., 2000; Nowak et al., 2003; Hasenstaub et al., 2005). On the other hand, the baseline firing rate difference between inhibitory interneurons and excitatory pyramidal neurons is a common finding throughout numerous studies related to classification of these two neuronal groups in the last two decades (Gur et al., 1999; Frank et al., 2001; Constantinidis and Goldman-Rakic, 2002; Maurer et al., 2006; Mitchell et al., 2007; Viskontas et al., 2007; Chen et al., 2008; Diester and Nieder, 2008; Le Van Quyen et al., 2008; Hussar and Pasternak, 2009, 2012; Johnston et al., 2009; Kuang et al., 2010; Yokoi and Komatsu, 2010; Tsunada et al., 2012; Woloszyn and Sheinberg, 2012; Kaufman et al., 2013). Thus, in the present study, we used not only the parameters of spike duration but also the baseline firing rate (spontaneous firing rate, SponT) to classify the neurons into BS neurons and NS neurons, namely putative pyramidal neurons and putative inhibitory interneurons, respectively. SponT was calculated from the neuron’s firing activities from 100 ms pre-stimulus onset to 300 ms post-stimulus onset, since stimulus velocity was still very small at 300 ms and the response was still kept at a baseline (for details, see Gu et al., 2006; Chen et al., 2010). Different neuronal groups were further sorted by the k-means algorithms (*k* = 3, squared Euclidean distance) using the two parameters: spike duration and SponT (Barthó et al., 2004; Sakata and Harris, 2009; Tsunada et al., 2012). We designated the cluster of neurons fell in long spike duration and low SponT as BS neurons and those fell in short spike duration and high SponT as NS neurons. Neurons that fell between those two clusters were called Unclassified in the present study. We could specify the number of clusters in K-means cluster analysis. As shown in Figures 2D,F, by setting the cluster number as 3, we could exclude the neurons having short spike durations but high SponTs to minimize false commissions as much as possible.

#### Analysis of Tuning Properties

**Figure 3:**
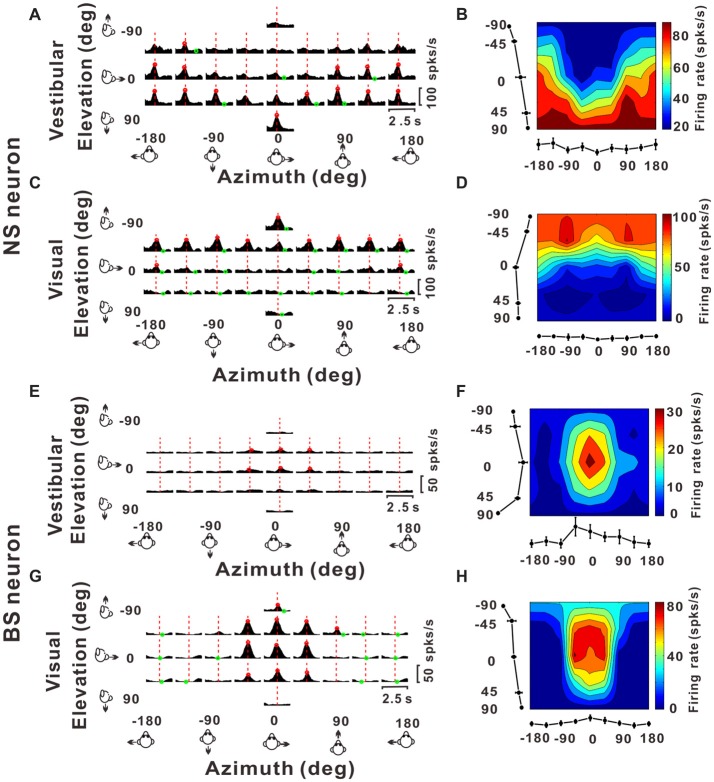
Example responses from a NS neuron and a BS neuron. **(A,C,E,G)** Response peristimulus time histograms (PSTHs) for an example NS neuron (**A** for vestibular stimuli and **C** for visual stimuli) and an example BS neuron (**E** for vestibular stimuli and **G** for visual stimuli). The averaged PSTHs for all 26 directions of vestibular or visual translation arranged according to stimulus direction in spherical coordinates. Vertical dashed red lines indicate the peak time of the neuron. PSTHs were computed with sequential 25 ms bins and then smoothed with a 400 ms sliding window (see “Materials and Methods” section). **(B,D,F,H)** Color contour maps of the neurons at the peak time. The peak time was taken from the preferred direction. Tuning curves along the bottom and left sides of each color contour maps show the 3D tuning at the peak time indicated by the red dashed lines in panels **(A,C,E,G)**. Noted that because azimuth 180° (away from azimuth 0° anticlockwise) and azimuth −180° (away from azimuth 0° clockwise) are overlapped on the horizontal plane, so in panels **(A,C,E,G)**, the left-most three panels were just copied from the right-most three panels for symmetry and actually there were only 26 directions out of the 29 panels.

To measure the direction tuning of the neurons, translational responses for visual or vestibular were measured along 26 directions in 3D (see “Experimental Protocol” section for detail). For each isolated single neuronal activity, we used and a 400 ms slide window stepped at 25 ms to construct a smoothed peristimulus time histograms (PSTHs) for each direction of translation. Mean firing rates during the 400 ms time window centered at the peak time were transformed using the Lambert cylindrical equal-area projection (Snyder, 1987), and then plotted as a function of azimuth and elevation in color contour maps to visualize 3D spatial tuning (Chen et al., 2011c; as shown in right panels in Figure 3). In these plots, the abscissa represents azimuth and the ordinate represents a cosine-transformed version of elevation.

#### Temporal Modulation Analysis

To test whether the temporal modulation along each stimulus direction was significant, we first identified the 400 ms time windows containing the spike count distributions having the maximum and/or minimum values. Then we compared these distributions with SponT distribution to see whether they are significantly different from each other (Wilcoxon signed rank test, *p* < 0.01, for details, see Chen et al., 2010). At least four overlapping time windows (spaced 25 ms apart and including the maximum or minimum time window defined above) were required to have spike count distributions that differed significantly from the baseline distribution to avoid false positives. For each stimulus condition and direction of movement, we used this statistical test to identify whether there are a significant peak and/or trough in the PSTH, otherwise the cell was deemed not to be responsive to stimulation in that direction.

#### Peak Times

As reported previously, times of the local maximum or minima at which distinct epochs of directional tuning happened were defined as “peak times” (for details, see Chen et al., 2010). First, for each 25 ms time bin between 0.5 s and 2 s after stimulus onset, the maximum/minimum response of the neuron across directions was calculated. Then the statistical significance of direction selectivity for each time bin was further evaluated by one-way ANOVA test (five repeats for each direction). By checking the statistical significance of direction tuning as a function of time, we decided whether there are multiple time periods in which a neuron shows distinct temporal peaks of directional tuning (see Chen et al., 2010). Cells without peak times were excluded from all the analyses in present study.

#### Preferred Direction

For each stimulus condition, the mean firing rate during the 400 ms window whose center aligned at peak response time in each trial was represented by the magnitude of a 3D vector whose direction defined by the elevation and azimuth angles (Gu et al., 2006). The preferred direction of this neuron under each condition was further computed from the vector sum’s elevation and azimuth of each neuron’s responses (spontaneous activity subtracted).

#### Half Tuning Width

To quantify the tuning characteristics of the neurons, we also constructed tuning width curves from the horizontal tuning curve. The mean firing rates of the neuron at its peak time in eight horizontal directions were shifted such that the maximum firing rate was aligned at 0°. Then the directional tuning curve was fitted by a Gaussian function, *r*(*θ*) (Chen et al., 2010):

$$r(\theta )=a\times \text{exp}\langle -2\{[1-\text{cos}(\theta -\theta_{\text{pref}})]/\sigma^{2}\}\rangle +b$$

where *θ*_pref_ indicates the preferred direction, *σ* represents the half peak width of the tuning curve, *a* indicates the tuning curve amplitude, and *b* denotes the baseline firing rate, *σ* is defined as the half tuning width.

#### Strength of Direction Tuning

The 3D directional tuning strength was defined using a direction discrimination index direction discrimination index (DDI), given by Takahashi et al. (2007):

$$DDI=\frac{R_{\max}-R_{\min}}{R_{\max}-R_{\min}+2\sqrt{SSE/(N-M)}}$$

*R*_max_ and *R*_min_ are the maximum and minimum firing rate from the 3D tuning function, respectively. *SSE* represents the sum squared error around the mean response, *N* is for the total number of trials, and *M* denotes the number of directions (*M* = 26). Thus, the reliability of a neuron for discriminating the preferred motion direction from the null motion direction can be quantified by *DDI*, whose value ranges from 0 to 1, corresponding to response modulations that range from weak to strong.

## Results

We recorded MSTd and VPS neurons as monkey was passively translated in physical (vestibular) or simulated (visual) motion along 26 motion direction uniformly distributed in 3D space (Figures 1A,B). The movement along each direction followed a Gaussian velocity profile, as shown in Figure 1C. The corresponding acceleration profile is biphasic. For each block, we randomly give monkey vestibular or visual stimulus. A null condition was also contained for control, and monkey was asked to fixate at the target of head-centered position without any vestibular or visual condition during this trial. To exclude any multiunit activity that may average the waveforms and blur differences in the population, we only included units that had been clearly isolated from noise and other units by offline spike sorting based on clustering in the principal components of waveforms on that electrode (Spike2, Cambridge Electronic Design). In total, 324 single units from MSTd (three rhesus monkey) and 97 single units from VPS (two rhesus monkey) were isolated. We further divided these units into putative interneurons (NS neurons) and putative pyramidal neurons (BS neurons) according to the parameters of spike duration and base line firing rate, then compared the spatiotemporal response characteristics to 3D translation between these two groups of neurons.

### Classification of Interneurons and Pyramidal Cells

An example of the classification of interneurons and pyramidal cells is shown in Figures 2A,B. Figure 2A shows 6 ms of filtered raw signals collected from one electrode. It is clear that there are two kinds of spike waveform beyond the noise. Thus, all the spikes from this electrode were further sorted into two isolated neurons according to the spike waveforms. The spikes recorded in each neuron were averaged after the alignment by troughs, and the mean waveforms (±1 SD) was shown in Figure 2B for two isolated neurons collected from the same electrode. In the current study, the spike duration of a neuron was calculated from the interval between the peak and trough of the mean spike waveform, as shown in the inserted figure at the top right corner in Figure 2B. Figures 2C,E show the mean spike waveforms from all the isolated MSTd and VPS neurons, respectively. Each curve representing the mean spike waveform from one neuron. From these figures, we can see that most of the recorded spike waveforms exhibited variations in duration although they have similar biphasic shape. It should be noted that the spike waveforms were normalized by the difference between their peak and trough values before calculating the spike durations. All except eight MSTd and one VPS neuron, exhibited in the inset, had waveforms with a negative trough followed by a clear positive peak. And these nine cells were excluded from further analysis.

Since the discharge frequencies and spike durations varied between pyramidal cells and interneurons (Mountcastle et al., 1969; McCormick et al., 1985; Rao et al., 1999; Constantinidis and Goldman-Rakic, 2002; Nowak et al., 2003; Likhtik et al., 2006), we used these two characters to discriminate principal excitatory units (putative pyramidal cells) and inhibitory units (putative interneurons; for details, see “Materials and Methods” section). As shown in Figures 2D, K-means cluster analysis (*k* = 3) was applied to the distribution of all isolated neurons’ spike durations and SponTs, and the neurons were separated into three clusters (see “Materials and Methods” section for more information). Neurons fell in long spike duration and low SponT were assigned to putative pyramidal neurons and those fell in short spike duration and high SponT were deemed putative interneurons. Neurons that fell between those two clusters were called “**Unclassified**” in the present study and were excluded from further analysis (Figures 2D,F). As a result, 55 neurons were classified as putative interneurons (Silhouette value = 0.43) and 90 neurons were classified as putative pyramidal neurons (Silhouette value = 0.45) in MSTd, while in VPS 11 putative interneurons (Silhouette value = 0.81) and 28 putative pyramidal neurons (Silhouette value = 0.82) were classified for the remaining analysis. Overall, the fraction of our recordings identified as NS putative interneurons among all the identified neurons (38% for MSTd; 28% for VPS) was similar to the fraction present in other physiological study (20%–30%; Connors and Gutnick, 1990).

### Temporal Modulation for Putative Interneurons and Pyramidal Neurons

**Figure 4:**
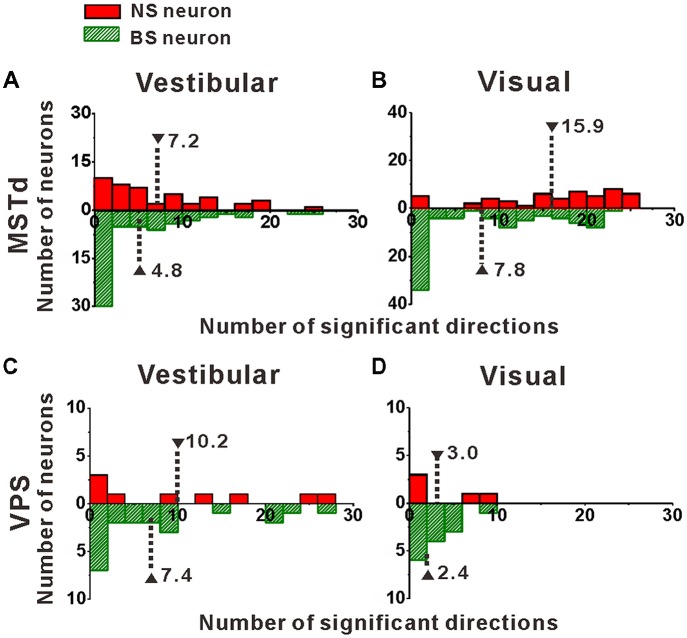
Distribution of the number of significant directions. **(A,B)** Distribution histogram of the number of neurons that have a significant response to vestibular **(A)** and visual stimuli **(B)** for NS and BS neurons in MSTd. A total of 45 NS neurons and 61 BS neurons with significant directional tune to vestibular stimuli were selected (both *p* < 0.05, ANOVA test) and a total of 52 NS neurons and 83 BS neurons with significant directional tune to visual stimuli were selected (both *p* < 0.05, ANOVA test). **(C,D)** Distribution histogram of the number of neurons that have a significant response to vestibular **(C)** and visual stimuli **(D)** for NS and BS neurons in VPS. The neurons analyzed in the graph have a significant directional tune to vestibular or visual stimuli (both *p* < 0.05, ANOVA test). Vestibular tuning: NS neuron (*N* = 13), BS neuron (*N* = 7). Visual tuning: NS neuron (*N* = 22), BS (*N* = 19). Note that the number of significant direction distributions for BS neurons were plotted as downward projecting histograms simply for illustrative purposes.

Once we classified the recorded responsive neurons into NS and BS neurons, we examined the 3D responses between these two neuronal groups under vestibular and visual stimuli. Example responses of a NS and a BS neuron from MSTd are illustrated in Figure 3. Figure 3A shows the averaged PSTHs for all 26 directions of vestibular translation arranged according to stimulus direction in spherical coordinates. The red circle represents a significant peak in that PSTH and the green circle indicates a significant trough in the PSTH. This NS neuron responds significantly to 13 motion directions during vestibular condition (Figure 3A) and 22 motion directions during visual condition (Figure 3C). These numbers are larger than that of the example BS neuron (*N* = 7 for vestibular stimuli and *N* = 19 for visual stimuli). The population summary of the number of significant responsive directions was shown in Figure 4. In MSTd, the number of significant responsive directions of NS neurons under the vestibular condition (Figure 4A, mean ± SE: 7.2 ± 1.0) was significantly higher than that of BS neurons (mean ± SE: 4.8 ± 0.8; *p* < 0.05, Wilcoxon rank-sum test). During visual condition, the difference was more obvious with the number of significant responsive directions being 15.9 ± 7.4 (mean ± SE) for NS neurons and 7.8 ± 0.9 (mean ± SE) for BS neurons (Figure 4B, *p* < 0.001, Wilcoxon rank-sum test). These results suggest putative interneurons are more broadly tuned by different directions across sphere than putative pyramidal neurons in MSTd. Weaker but similar results were observed in VPS, as shown in Figures 4C,D. Although there were no significant differences between these two classes of neurons (vestibular: *p* = 0.56; visual: *p* = 0.99, Wilcoxon rank-sum test), the average number of significant responsive directions for NS neurons (mean ± SE: 10.2 ± 3.5 in vestibular condition; 3.0 ± 1.8 in visual condition) was still larger than that for BS neurons (mean ± SE: 7.4 ± 1.8 in vestibular condition; 2.4 ± 0.7 in visual condition). However, in contrast to MSTd, the broader direction tuning of putative interneurons are more obvious for vestibular stimuli rather for visual stimuli in VPS. This might be caused by that MSTd is dominated by visual signals while VPS is dominated by vestibular signals.

### 3D Spatial Tuning for Putative Interneurons and Pyramidal Neurons

Above results showed that NS neurons were responsive to more direction in 3D. To address whether the larger number of significant responsive directions was caused by selective stronger inputs or evenly elevated responses along all the directions, we further quantified the neurons’ 3D spatial tuning properties.

Figures 3B,D illustrate 3D tuning at the peak time by the color contour maps for the example NS neuron from MSTd under vestibular and visual stimuli, respectively. Figures 3F,H show an example of BS neuron. It should be noted that peak time that produces the largest firing rate comparing to the baseline response, is indicated by the red dashed lines in Figure 3A; and the 3D directional tuning of this neuron at this peak time (0.93 s) to the vestibular stimuli was shown as the color contour map in Figure 3B. In this map, mean firing rate (represented by color) is plotted as a function of azimuth and elevation. This neuron was significantly tuned during vestibular translation (one-way ANOVA for firing rates across 26 directions with five repeats for each direction, *p* < 0.001) and exhibited broad tuning with a preferred direction at 75° azimuth and 90° elevation, corresponding to a motion downward. For the visual condition, this neuron was also broadly tuned (one-way ANOVA for firing rates across 26 directions with five repeats for each direction, *p* < 0.001) at its peak time (1.18 s), with a preferred direction at 0° azimuth and −90° elevation (Figure 3D), corresponding to an upward motion trajectory. In contrast, the example BS neuron exhibited relatively sharp tuning for both vestibular and visual translation (one-way ANOVA for firing rates across 26 directions with five repeats for each direction, *p* < 0.001), with a preferred direction at 0° azimuth and 0° elevation, corresponding to a forward motion trajectory in horizontal plane under vestibular stimuli (Figure 3F, at the peak time of 1.45 s) and a preferred direction at 0° azimuth and −23° elevation, corresponding to a forward and slightly upward motion trajectory under visual stimuli (Figure 3H, at the peak time of 1.28 s). The example NS MSTd neuron was tuned more broadly than the example MSTd BS neuron both under vestibular and visual stimuli. To see whether this was a common difference between classes of NS and BS neurons, we further compared the tuning width distribution between these two groups of neurons in the following analyses.

**Figure 5:**
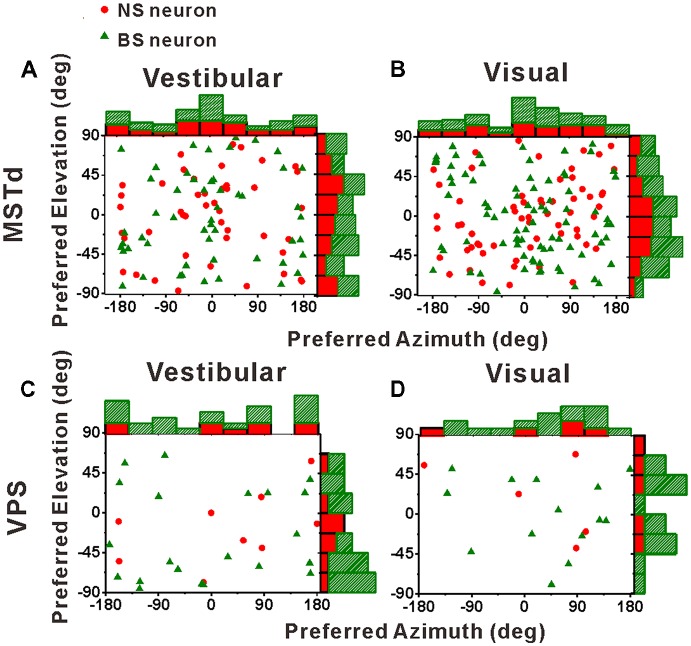
Distributions of 3D heading preferences for NS (red) neurons and BS (green) neurons in areas MSTd **(A,B)** and VPS **(C,D)** in response to vestibular and visual stimuli. Each data point in the scatter plot corresponds to the preferred azimuth (abscissa) and elevation (ordinate) of a single neuron with significant heading tuning. Histograms along the top and right sides of each scatter plot show the marginal distributions. The neurons analyzed in the graph have a significant directional tune to vestibular or visual stimuli (both *p* < 0.05, ANOVA test). The number of neurons were same as in Figure 4.

Before comparing the tuning widths, we first looked at the distributions of the preferred directions of the population NS neurons and BS neurons across the spherical stimulus space, as shown in Figure 5. For both NS and BS neurons in MSTd, the preferred directions were distributed throughout the spherical stimulus space under either vestibular stimulus (Figure 5A) or visual stimulus (Figure 5B). Situation in VPS shown was same as in MSTd, as shown in Figures 5C,D for vestibular and visual condition, respectively. The preferred directions of the NS and BS neurons in VPS were also widely distributed throughout the spherical stimulus space in both stimulus conditions. All the distributions fitted the normal distribution (Shapiro-Wilk test, the minimal value of *p* was 0.36) and there is no clear difference for the direction preferences between interneurons and pyramidal neurons (*T*-test, the minimal value of *p* was 0.18).

**Figure 6:**
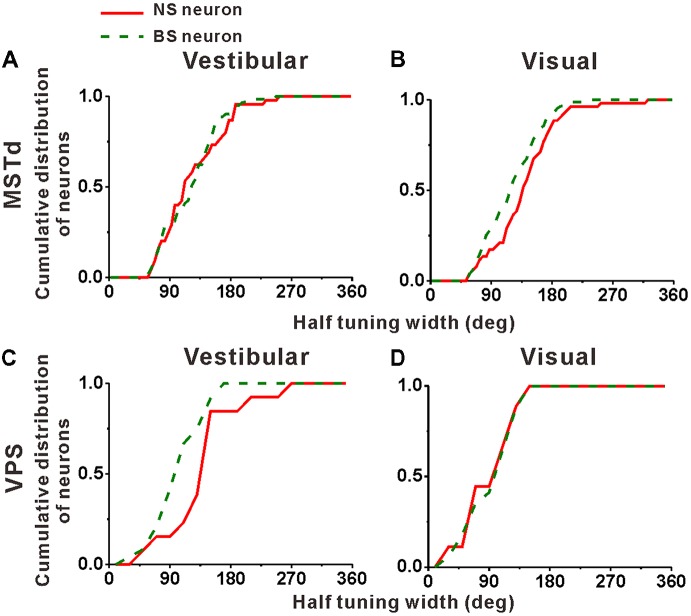
Population summary of the half tuning width of the NS (red) neurons and BS (green) neurons in areas MSTd **(A,B)** and VPS **(C,D)** under the vestibular and visual conditions. Cumulative frequency distribution of the neurons on the half tuning widths, which were computed from each neuron’s tuning curve in the horizontal plane, are given by the curves. The neurons analyzed in the graph have a significant directional tune to vestibular or visual stimuli (both *p* < 0.05, ANOVA test). The number of neurons were same as in Figure 4.

Cumulative distributions of the half tuning widths in MSTd are summarized for vestibular and visual condition in Figures 6A,B, respectively. For vestibular condition (Figure 6A), the cumulative curve of the NS and BS neurons were nearly overlapping, indicating that in terms of tuning width, no significant difference between these two classes of neurons was observed (mean ± SE: 125.3° ± 7.0° for NS; 123.2 ± 5.4° for BS; *p* = 0.61, Kolmogorov–Smirnov test). However, the distributions for these two groups of neurons during visual condition showed obvious tendency to be different (*p* = 0.09, Kolmogorov–Smirnov test). As shown in Figure 6B, the cumulative curve of the MSTd NS neurons rises slowly than that of the BS neurons in visual condition, which means that the NS neurons had broader tuning width (mean ± SE: 140.4 ± 6.9°) than the MSTd BS neurons (mean ± SE: 121.8 ± 4.5°). In contrast, for VPS neurons, the difference of the tuning width for these two group neurons was observed in vestibular condition rather than visual condition, as shown in Figures 6C,D, respectively. In vestibular condition, the tuning width was obviously (although not very significantly) larger in NS neurons (mean ± SE: 156.2 ± 19.3°) than in BS neurons (mean ± SE: 113.5 ± 7.4°; *p* = 0.05, Kolmogorov–Smirnov test). However, in visual condition, these two distributions are almost overlapping, and the tuning widths are not significantly different from each other (mean ± SE: 101.3 ± 14.8° for NS neurons; 106.1 ± 9.2° for BS neurons; *p* = 0.87, Kolmogorov–Smirnov test). One possible explanation for the difference between MSTd and VPS is that the putative interneurons are more broadly tuned than the putative pyramidal neurons when they response to their dominant stimuli rather than non-dominant stimuli.

All the above analysis seems to demonstrate that the NS neurons showed more broadly direction tuning than BS neurons, Intuitively, the broader tuning curve might cause weaker tuning strength. To examine whether it is true, we further compared the direction tuning strength between the NS and BS neurons by using DDI (for details, see “Strength of Direction Tuning” in “Data Analysis” section and Takahashi et al., 2007). It can quantify the reliability of a neuron for discriminating the preferred motion direction from the null motion direction. The value of DDI ranges from 0 to 1, 0 represents the weakest direction discrimination, and one represents the strongest directional discrimination.

**Figure 7:**
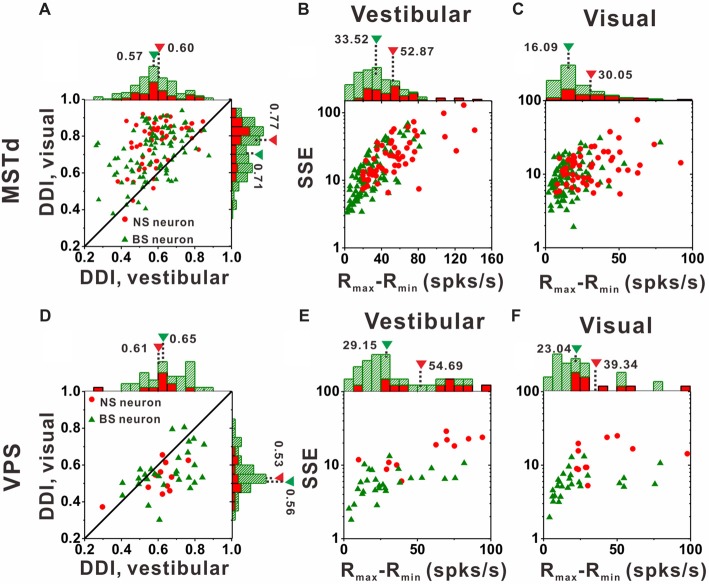
Tuning strengths to the NS (red) neurons and BS (green) neurons in areas MSTd (upper panel) and VPS (lower panel) under the vestibular and visual conditions. **(A)** Scatter plots of direction discrimination index (DDI) values for the vestibular (abscissa) and visual (ordinate) conditions, respectively. **(B,C)** Scatter plots of each neuron’s firing rate variabilities (standard deviation, SD) over the maximum difference of the neuron’s firing rates (R_max_ − R_min_) for the vestibular condition **(B)** and the visual condition **(C)**. The histograms on the top and right show the marginal distributions. **(D–F)** Same as **(A–C)** respectively but for area VPS.

Figure 7A compares DDI values of the MSTd between NS neurons and BS neurons. For both NS (green) and BS (red) neurons, the majority of them were distributed above the diagonal, indicating that DDI values under visual condition are larger than that under vestibular condition (both *p* < 0.001, Wilcoxon rank-sum test) for both groups. The averaged DDI values were 0.60 ± 0.01 (mean ± SE) for NS neurons and 0.57 ± 0.01 (mean ± SE) for BS neurons in vestibular condition. There was no significant difference between these two groups (*p* = 0.55, Wilcoxon rank-sum test). However, the DDI values of the NS neurons was significantly larger than that of the BS neurons (mean ± SE: 0.77 ± 0.02 for NS neurons; mean ± SE: 0.71 ± 0.01 for BS neurons, *p* < 0.01, Wilcoxon rank-sum test) in visual condition. As larger DDI values usually indicate the stronger directional discrimination ability, the higher DDI values for NS neurons seems to be conflict with the broader tuning results. One possible explanation is that wider tuning curve was caused by the combination results of flat tuning curve and smaller response variability across different directions. Since DDI was affected both by R_max_ − R_min_ (the difference between the maximum and the minimum firing rate across 26 directions) and SSE (the sum of squared error of the firing rates in 26 directions), the larger DDI values of the NS MSTd neurons in visual condition might be resulted from larger R_max_ − R_min_ or smaller SSE. To examine it, Figures 7B,C compared the distribution of R_max_ − R_min_ and SSE between the NS and BS neurons in vestibular and visual condition, respectively. For both vestibular and visual conditions, the distribution of the red dots (representing the NS neurons) was more inclined to have the larger values along the horizontal and longitudinal axis than that of the green triangles (represent the BS neurons). In the vestibular condition, the averaged response amplitude of the NS neurons (mean ± SE: 52.9 ± 4.1, spikes/s) was significantly higher than that of the BS neurons (mean ± SE: 33.5 ± 2.4; *p* < 0.01, Wilcoxon rank-sum test), meanwhile the SSE of the NS neurons (mean ± SE: 26.9 ± 3.4) was also significantly larger than that of the pyramidal neurons (mean ± SE: 18.8 ± 2.1; *p* < 0.01, Wilcoxon rank-sum test). Similar results were observed in the visual condition (Figure 7C): the R_max_ − R_min_ of the NS neurons (mean ± SE: 30.0 ± 2.5) was significantly higher than that of the BS neurons (mean ± SE: 16.1 ± 1.3; *p* < 0.01, Wilcoxon signed rank test), and the SSE of the NS neurons (mean ± SE: 13.2 ± 0.9) was also significantly larger than that of the BS neurons (mean ± SE: 9.9 ± 0.6; *p* < 0.01, Wilcoxon rank-sum test). Thus, the larger DDI values of the MSTd NS neurons for visual stimuli was mostly attributed to larger modulation depth compared with the response variability (SSE). This had no causal link to flat tuning curve or smaller response variability across different directions that account for broader tuning curves of MSTd NS neurons, thus the challenge of conflict did not exist here.

In contrast to MSTd, VPS is a vestibular-dominant area. Consequently, the direction tuning strength of both NS neurons and BS neurons for vestibular condition was significantly greater than those for visual condition (Figure 7D, *p* < 0.001, Wilcoxon rank-sum test). For both vestibular and visual condition, the DDI values of NS neurons were not significantly different from BS neurons, with averaged values 0.61 ± 0.04 (mean ± SE) for NS and 0.65 ± 0.02 (mean ± SE) for BS neurons during vestibular condition(*p* = 0.37, Wilcoxon rank-sum test) while 0.53 ± 0.03 (mean ± SE) and 0.56 ± 0.02 (mean ± SE) for NS and BS neurons during visual condition (*p* = 0.36, Wilcoxon rank-sum test). However, the distribution of the R_max_ − R_min_ and SSE between these two groups of neurons in VPS showed the same tendency as in MSTd. For vestibular condition (Figure 7E), the R_max_ − R_min_ of the NS neurons was significantly higher than that of the BS neurons (mean ± SE: 54.7 ± 20.9 vs. 29.2 ± 5.1, *p* < 0.01, Wilcoxon rank-sum test). The SSE of the NS neurons was also significantly larger than that of the pyramidal neurons (mean ± SE: 16.6 ± 6.1 vs. 6.2 ± 1.3, *p* < 0.01, Wilcoxon rank-sum test). Results were similar for visual condition (Figure 7F): both the R_max_ − R_min_ and SSE of the NS neurons were significantly higher than that of the BS neurons (*p* < 0.01, Wilcoxon rank-sum test). The averaged R_max_ − R_min_ was 39.3 ± 13.1 (mean ± SE) for NS and 14.3 ± 6.4 (mean ± SE) for BS neurons, respectively, and the averaged SSE was 14.3 ± 6.4 (mean ± SE) for NS and 5.9 ± 1.5 (mean ± SE) for BS neurons respectively. Since in MSTd, the DDI values of NS neurons was significantly larger than that of BS neurons in visual condition, it was natural to expect that in VPS, the DDI values of the NS neurons was significantly larger than that of BS neurons in vestibular condition, however, it was not the case. We noted that the VPS neurons’ R_max_ − R_min_ and SSE were obviously larger than that of the MSTd, which indicate the relatively poorer and more diverse response of the VPS neurons upon stimulation and may account for the difference between MSTd and VPS.

### Comparison of Peak Times Between Narrow-Spiking and Broad-Spiking Neurons

**Figure 8:**
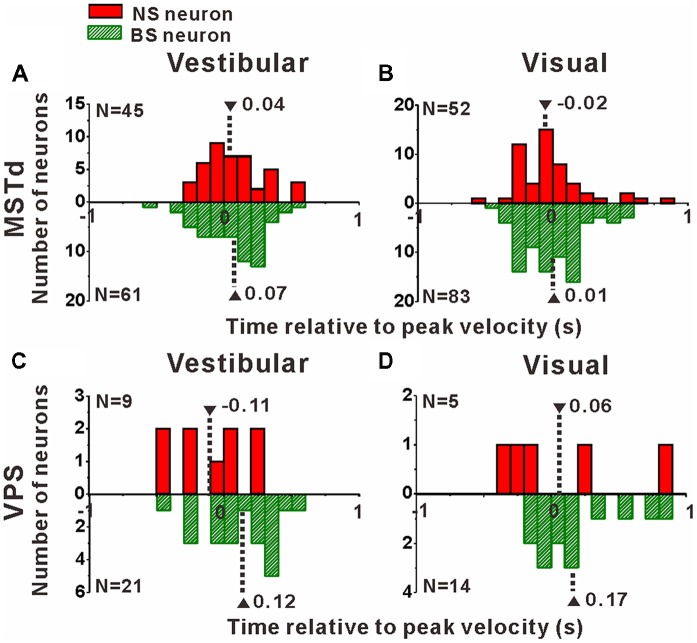
Peak times of the NS (red) neurons and BS (green) neurons in areas MSTd **(A,B)** and VPS **(C,D)** under the vestibular and visual conditions. The neurons analyzed in the graph have a significant directional tune to vestibular or visual stimuli (both *p* < 0.05, ANOVA test). The number of neurons were same as in Figure 4. Note that the number of significant direction distributions for BS neurons were plotted as downward projecting histograms simply for illustrative purpose.

Interneurons are supposed to modulate the activities of output pyramidal neurons in local circuitry to fulfill information transmission to next stage through feedforward or feedback connections (Wilson, 2007; Suzuki and Bekkers, 2012), thus the response latency of the putative pyramidal and interneurons might be different from each other. In the present study, motion stimuli were dynamic and followed a Gaussian velocity profile with peak velocity of 30 cm/s at 1 s. The recorded neurons were not activated and kept the baseline response during the first several hundred milliseconds of the stimulus until the velocity increased large enough, and reached their peak firing rates around or after the timing of the peak velocity of the stimulus. So, it is impossible to measure the response latency of the neurons accurately in the present study, but we could measure the peak time of the response relative to the peak velocity of the stimulus instead. To test whether the peak times of the putative pyramidal and interneurons are different from each other, we further looked into the peak time distributions of these two neuronal populations as shown in Figure 8. In MSTd, peak times under vestibular stimuli averaged 0.04 ± 0.04 s (mean ± SE) relative to the peak velocity of the stimulus for NS neurons were significantly earlier than that for BS neurons (mean ± SE: 0.07 ± 0.03 s; Figure 8A, *p* < 0.05, Wilcoxon rank-sum test), meanwhile peak times under visual stimuli averaged —0.02 ± 0.04 s (mean ± SE) for NS neurons were also obviously earlier than that for BS neurons (mean ± SE: 0.01 ± 0.03 s; Figure 8A, *p* < 0.05, Wilcoxon rank-sum test). In VPS, peak times under vestibular stimuli averaged − 0.11 ± 0.10 s (mean ± SE) for NS neurons were earlier than that for BS neurons (mean ± SE: 0.12 ± 0.06 s; Figure 8C, *p* < 0.05, Wilcoxon rank-sum test). In visual condition, although peak times for NS neurons (mean ± SE: 0.06 ± 0.22 s) showed no significant difference from that for BS neurons (mean 26± SE: 0.17 ± 0.05 s; Figure 8D, *p* = 0.46, Wilcoxon rank-sum test), the mean value of NS neurons was still smaller than that of BS neurons. This result showed that in self-motion perception, the putative interneurons in the local circuits for both MSTd and VPS areas may receive the external information earlier than the putative pyramidal neurons, may suggesting feedforward modulation of the putative interneurons to pyramidal neurons.

## Discussion

We classified MSTd and VPS neurons into putative interneurons with narrow spikes and putative pyramidal neurons with broad spikes, then compared the spatiotemporal direction tuning properties between these two classes. Our main findings are that putative interneurons responded to visual or vestibular signals with earlier peak times, higher firing rates and greater variability, and the broader tuning of putative interneurons were more obviously observed for visual condition in MSTd and vestibular condition in VPS. These findings suggest a general functional distinction between inhibitory and excitatory neurons across cortex.

### Comparison With Previous Neuron Classification

The fraction of our recordings identified as NS putative interneurons among all the identified neurons (38% for MSTd; 28% for VPS) was close to but a little bigger than the fraction present in an early physiological study (20%–30; Connors and Gutnick, 1990) and several previous studies that reported small proportions (10%–30) of NS neurons (Wilson et al., 1994; Markram et al., 2004; Mitchell et al., 2007; Atencio and Schreiner, 2008; Diester and Nieder, 2008; Isomura et al., 2009; Sakata and Harris, 2009; Yokoi and Komatsu, 2010; Ison et al., 2011). One possible reason is the cell classification criteria. Instead of using spike duration as the sole criteria as they do in some of the previous classifications (Barthó et al., 2004; González-Burgos et al., 2005; Krimer et al., 2005; Merchant et al., 2008; Le Van Quyen et al., 2008; Isomura et al., 2009), we used two parameters in the present study, spike durations (i.e., the trough-to-peak times) and baseline firing rates to classify the NS and BS neurons. Intracellular studies have demonstrated that GABAergic inhibitory interneurons produce spikes of much shorter duration than glutamatergic excitatory pyramidal neurons (McCormick et al., 1985; Connors and Gutnick, 1990; Nowak et al., 2003). The metric of spike duration, although there is still much place for perfection, appears to work reasonably well especially for extracellular recordings because: (1) the heterogeneous nature that lies in inhibitory interneurons (Kawaguchi and Kubota, 1997; Markram et al., 2004); it is a group in which some of them exhibit intermediate (González-Burgos et al., 2005; Krimer et al., 2005; Brill and Huguenard, 2009) or even broad (Merchant et al., 2008) waveforms, while spike waveforms exhibited by some of the neurons that tract to pyramidal cells can also be very narrow, which resembles inhibitory neurons (Vigneswaran et al., 2011); and (2) in extracellular recordings, spike durations have been demonstrated to vary, as much as 0.7 ms, with the distance between the exact position of the electrode and the recorded cell (Likhtik et al., 2006). It was shown in the rat hippocampus that, the combined criterion of three factors: baseline firing rate, duration of spikes and bursting characteristics, enabled the effective separation of cells into interneurons and pyramidal neurons (Ranck, 1973; Csicsvari et al., 1999), which has been further supported by *in vivo* intracellular labeling and through recording simultaneously from the inside and outside of neurons of the same types (Henze et al., 2000; Klausberger et al., 2003) and even more studies support this by concurrent identification of either excitatory projection neurons through a method called antidromic stimulation (Likhtik et al., 2006; Johnston et al., 2009) or inhibitory interneurons by means of pairwise recordings and cross-correlation analysis (Barthó et al., 2004; Tamura et al., 2004). Additionally, the baseline firing rate difference between inhibitory interneurons and pyramidal neurons is a common exhibition throughout studies related with classification (Gur et al., 1999; Frank et al., 2001; Constantinidis and Goldman-Rakic, 2002; Maurer et al., 2006; Mitchell et al., 2007; Viskontas et al., 2007; Chen et al., 2008; Diester and Nieder, 2008; Le Van Quyen et al., 2008; Hussar and Pasternak, 2009; Johnston et al., 2009; Kuang et al., 2010; Yokoi and Komatsu, 2010; Tsunada et al., 2012; Woloszyn and Sheinberg, 2012; Kaufman et al., 2013). So in our study, we combined the criteria of spike duration and baseline firing rate to classify these two neuronal groups and excluded equivocal neurons between these two classes to minimize false commissions as much as possible. There may be some omission, but it will not alter the present findings.

### Comparison With Previous Studies Testing Properties of Narrow-Spiking and Broad-Spiking Neurons

Our finding of broader tunings for NS neurons than BS neurons in MSTd and VPS during translational self-motion is consistent with previous studies of differential functional role for interneurons and pyramidal neurons in vision, audition, somato sensation and motor control (Wilson et al., 1994; Swadlow, 2003; Markram et al., 2004; Mitchell et al., 2007; Viskontas et al., 2007; Zoccolan et al., 2007; Diester and Nieder, 2008; Isomura et al., 2009; Johnston et al., 2009; Yokoi and Komatsu, 2010; Ison et al., 2011; Mruczek and Sheinberg, 2012; Tsunada et al., 2012). The broader tunings of putative interneurons were also found in monkey prefrontal cortex for spatial tuning (Constantinidis and Goldman-Rakic, 2002) and for number representations (Diester and Nieder, 2008), in cat primary visual cortex for orientation (Cardin et al., 2007) and direction representation (Nowak et al., 2008), in mouse primary visual cortex for orientation and spatial frequency tuning (Sohya et al., 2007; Niell and Stryker, 2008; Liu et al., 2009; Kerlin et al., 2010; Runyan et al., 2010), in mouse auditory cortex for frequency tuning (Lin and Liu, 2010), in the primate motor system for directional tuning (Merchant et al., 2008) and in primate inferior temporal cortex for images representation (Mruczek and Sheinberg, 2012).

The shorter response latencies (corresponding to our finding of earlier peak times) of putative interneurons were as well universal in some of these studies mentioned above and other studies across cortex areas and species (Wilson et al., 1994; Diester and Nieder, 2008; Merchant et al., 2008; Hussar and Pasternak, 2009; Lin and Liu, 2010; Yokoi and Komatsu, 2010; Murray and Keller, 2011; Mruczek and Sheinberg, 2012). One related question is why inhibitory interneurons fire earlier? There are several speculations: (1) maybe for the simple reason that a lower activation threshold is possible for inhibitory neurons (Connors and Gutnick, 1990) because of low-threshold Na+ channel subtypes (Li et al., 2014), which can also account for the higher spontaneous rates, since spiking initiation may requires very little input; (2) preferential input produced by “fast” signal pathways (Hernández-González et al., 1994; Chen et al., 2007) or top-down inputs oriented from prefrontal cortex (Bar, 2003) may be received by inhibitory neurons, both of which would likely convey coarser responses of less selectivity; whereas the traditional information conducting stream hierarchy may be the dominant input to excitatory neurons; and (3) it is possible that the recording positions of the inhibitory interneurons and excitatory pyramidal neurons were in different layers. Therefore, the peak time differences between putative inhibitory interneurons and pyramidal neurons could be explained by a local processing hierarchy.

### Regional Differences and Network Implications for Self-Motion Perception

Although both MSTd and VPS putative interneurons show broader tuning and earlier peak times than putative pyramidal neurons, the differences between these two classes of neurons were not identical in MSTd and VPS. First, in MSTd, broader direction tunings of putative interneurons were found for visual stimuli but not for vestibular stimuli, while in VPS, putative interneurons were more broadly tuned to directions in vestibular condition instead of visual condition. Previous studies have demonstrated that MSTd area is dominated by visual stimuli and VPS area is dominated by vestibular stimuli although these two areas are multisensory for visual and vestibular self-motion stimuli (Gu et al., 2006; Chen et al., 2011b). Thus, we speculate that regional diversity of tuning width differences of the putative interneurons and pyramidal neurons between MSTd and VPS areas is very likely due to their dominant inputs. Second, significantly larger tuning strengths (defined as DDI values) of putative interneurons were only found in MSTd in visual condition. Analysis of the neurons’ R_max_ − R_min_ and SSE showed that the larger DDI values of MSTd putative interneurons in visual condition was mostly due to the greater discrepancy between the neurons’ R_max_ − R_min_ and SSE in visual condition as MSTd area is visual-dominant, while greater DDI values of VPS putative interneurons in vestibular condition did not occur because of the relatively poorer and more diverse response of the VPS neurons upon stimulation, reflected by the larger SE of the VPS neurons’ R_max_ − R_min_ and SSE. However, the larger SSE might be resulted from the smaller amount of VPS neurons in present study, thus it is too hasty to reject the possibility of the expectation that VPS putative interneurons may also have larger DDI values in vestibular condition. Thus so far, the regional differences across MSTd and VPS areas are mainly due to the difference of the dominant inputs.

Besides slight diversity between MSTd and VPS due to the difference of stimulus dominance and response variation, results in present study clarify the general points in basic response characteristics that differ in inhibitory and excitatory neurons in self-motion. Although the classification of neurons into putative inhibitory interneurons and pyramidal neurons is oversimplified and the circuit mechanism still cannot be elucidated, it has important implications for us to understand the role these neuron classes play in self-motion perception. Self-motion perception is complicated and involves several cortical areas with diverse functions. What roles do different classes of neurons play in the whole network? Further studies extending to identify, position and trace different neuronal classes, and analyzing their functional properties will be useful and critical for understanding the neural mechanism underlying self-motion perception and information transmission across cortical areas.

## Author Contributions

AC designed research. YZ, SL and DJ performed research. YZ and SL analyzed data. YZ, SL and AC wrote the article.

## Conflict of Interest Statement

The authors declare that the research was conducted in the absence of any commercial or financial relationships that could be construed as a potential conflict of interest.
